# Regulating the electronic structures of mixed B-site pyrochlore to enhance the turnover frequency in water oxidation

**DOI:** 10.1186/s40580-022-00311-z

**Published:** 2022-05-18

**Authors:** Cheng Zhang, Fangfang Wang, Beichen Xiong, Hong Yang

**Affiliations:** 1 Department of Chemical and Biomolecular Engineering, University of Illinois at Urbana-Champaign, 600 S. Mathews Avenue, 61801 Urbana, IL USA; 2 School of Mechanical and Power Engineering, Zhengzhou University, 100 Science Avenue, 450001 Zhengzhou, Henan People’s Republic of China

**Keywords:** Oxygen evolution reaction, Pyrochlore, Metal substitution, Turnover frequency, Hydroxyl group, Electronic structure

## Abstract

This paper describes the development of mixed B-site pyrochlore Y_2_MnRuO_7_ electrocatalyst for oxygen evolution reaction (OER) in acidic media, a challenge for the development of low-temperature electrolyzer for green hydrogen production. Recently, several theories have been developed to understand the reaction mechanism for OER, though there is an  uncertainty in most of the cases, due to the complex surface structures. Several key factors such as lattice oxygen, defect, electronic structure, oxidation state, hydroxyl group and conductivity were identified and shown to be important to the OER activity. The contribution of each factor to the performance however is often not well understood, limiting their impact in guiding the design of OER electrocatalysts. In this work, we showed mixed B-site pyrochlore Y_2_MnRuO_7_ catalyst exhibits 14 times higher turnover frequency (TOF) than RuO_2_ while maintaining a low overpotential of ~ 300 mV for the entire testing period of 24 h in acidic electrolyte. X-ray photoelectron spectroscopy (XPS) analysis reveals that this B-site mixed pyrochlore Y_2_MnRuO_7_ has a higher oxidation state of Ru than those of Y_2_Ru_2_O_7_, which could be crucial for improving OER performance as the broadened and lowered Ru 4d band resulted from the B-site substitution by Mn is beneficial to the OER kinetics.

**Supplementary information:**

The online version contains supplementary material available at 10.1186/s40580-022-00311-z.

## Introduction

Development of advanced oxygen evolution reaction (OER) electrocatalysts is essential to the rapidly increasing requirement in energy storage through green hydrogen generation and metal-air batteries [1–17]. While OER catalysts are often studied in alkaline electrolytes, there are increasing interests in developing electrocatalysts in acidic media to take the advantages of fast proton conduction in polymer electrolyte membrane (PEM)-based water electrolysis systems. Production of green hydrogen under acidic condition is preferred when high ionic conductivity, good compactness, and fewer side reactions are taken into consideration in an electrolyzer [18–26]. While OER activity can be improved by varying the compositions, current electrocatalysts tend to exhibit poor performance. Noble metal based RuO_2_ and IrO_2_ continue to play important roles in acidic OER catalysts for industrial applications, despite their high price and low Earth’s crust reserve [9, 19, 20, 27, 28]. Thus, understanding the structures of electrocatalysts with both high activity and stability is needed.

Ruthenium and iridium based pyrochlores (A_2_B_2_O_7_) have been developed as promising OER electrocatalysts in acidic electrolyte for their high catalytic activity and good structural stability in acid [29–34]. We previously identified pyrochlore Y_2_Ru_2_O_7_ as one of the stable OER catalysts under low current density regime [20]. Subsequent work further enhanced the performance of Y_2_Ru_2_O_7_ through metal doping or defect engineering [35–38]. While regulating electronic structure through A-site doping has been studied, the B-site substitution is quite useful in the design of proper structures for high activity as well. Adding non-Ir or Ru elements may also reduce the use of precious metals, potentially resulting in high precious metal-based mass specific activity and lowered cost. With fewer Ru or Ir atoms per unit cell, the turnover frequency (TOF), which is the specific activity per unit mass of a precious metal, should be even higher than the one without the B-site substitution of Ru atoms [39]. For example, we developed porous, mixed B-site Y_2_[Ru_1.6_Y_0.4_]O_7_ pyrochlore as an acidic OER catalyst, which exhibited around eight times higher in TOF than Y_2_Ru_2_O_7_ [40].

Since OER catalysts are typically made of oxides (i.e., ceramic) at high temperature, formation of micron-sized particles or structures due to sintering often occurs. One strategy for improving the activity thus is to prepare nanometer (nm)-scaled catalysts, which may exhibit higher electrochemical active surface area and faster reaction kinetics than their bulk counterparts. For example, we previously prepared pyrochlore Y_2_Ru_2_O_7_ with a particle size of around 40 nm using polymer entrapment flash pyrolysis method, and showed an enhanced OER activity compared with those having larger sizes [31]. This strategy is complementary to those that affect the intrinsic reaction kinetics, such as oxidation state, conductivity, electronic structure, binding groups on the surface and fermi level, through metal substitution [35–37, 40, 41], to further improve the OER catalysts.

On the effects of structure on intrinsic OER kinetics, several mechanisms such as adsorbate evolution mechanism (AEM) and lattice oxygen mediated mechanism (LOM) were proposed to account for the observed relationships between the oxide structure and OER performance [26, 35, 42–44]. LOM suggests that the lattice oxygen in the electrocatalysts also participate the reaction, leading to a higher activity and lower kinetics barrier in quite a few complex oxides, when compared to the effects of AEM. It is noteworthy to point out that in both mechanisms the adsorption of hydroxyl group on surface is a crucial step, and the concentration of surface hydroxyl group was important for the OER performance [3, 45–48]. Thus, producing high concentration of hydroxyl groups on surface of nanometer (nm)-scaled complex oxides is a holistic strategy to improve the activity and stability of OER catalysts. In addition, the electronic structure and oxidation state of the metal center (i.e., Ru) is also important for the OER activity [49–51]. Nonstoichiometry of the metal ion, that is higher or lower than the standard valence, + 4 in the case of Ru cation, is thought to be beneficial to the fast kinetics of OER. Creating mixed B-site pyrochlores to regulate surface hydroxyl group concentration and Ru valence could be an effective way to generate the desired electronic structures with high catalytic performance.

In this paper, we describe the preparation of mixed B-site Y_2_[Mn_0.5_Ru_0.5_]_2_O_7_ (YMRO) compounds to regulate the surface properties, oxidation state and electronic structures for efficient OER activity in acidic media. This catalyst was designed based on the analyses above for the optimal TOF on a precious metal base, that is, (1) controlling the size in the nm-scaled regime, (2) using Mn to create nonstoichiometric compound and reducing the Ru usage per unit formula, (3) changing the oxidation state of Ru, and (4) regulating the surface density of hydroxyl groups through defect engineering (on B-site). Our results indicate that the YMRO exhibited much higher TOF value than Y_2_Ru_2_O_7_ (YRO) and the reference RuO_2_ despite YRO had a relatively higher surface density of hydroxyl groups. The YMRO electrocatalysts also maintained a stable low overpotential at a current density of 10 mA/cm^2^ for over 24 h in a chronopotentiometry study. X-ray photoelectron spectroscopy (XPS) analysis indicates that oxidation state of Ru increases after the B-site metal substitution, which could be a key contributing factor for the observed enhanced OER performance, suggesting the electronic configuration plays an important role in this mixed B-site pyrochlore OER catalysts.

## Methods/experimental

### Materials

Yttrium (III) nitrate hexahydrate (Y(NO_3_)_3_·6H_2_O, 99.9%), ruthenium (III) nitrosyl nitrate solution (Ru(NO)(NO_3_)_x_(OH)_y_, x + y = 3), Nafion^®^ 117 solution (~ 5%), ruthenium (IV) oxide (RuO_2_, 99.9%), and sodium carbonate monohydrate (Na_2_CO_3_·H_2_O, 99.5%) were purchased from Sigma-Aldrich. Iridium (IV) oxide (IrO_2_, 99.9%), manganese (II) nitrate tetrahydrate (Mn(NO_3_)_2_·4H_2_O, 99.9%), iridium(III) chloride hydrate (IrCl_3_·xH_2_O, 99%), and manganese(IV) oxide (MnO_2_, 99.9%) were purchased from Alfa Aesar. Citric acid monohydrate (C_6_H_8_O_7_·H_2_O, 99%) and sodium hydroxide (NaOH, 99.4%) were from Fisher Chemical. Veritas^®^ double distilled perchloric acid (HClO_4_, 70%) was obtained from GFS Chemicals. Yttrium(III) chloride hexahydrate (YCl_3_·6H_2_O, 99.9%) was purchased from Stream Chemicals. Tetrahydrofuran (THF) was obtained from Macron Fine Chemicals. Vulcan carbon XC-72 was purchased from Cabot Corporation. Hydrogen (H_2_, 99.999%) and oxygen (O_2_, 99.999%) were supplied by Airgas, Inc. All these chemicals and gases were used without further purification.

### Synthesis of mixed b-site pyrochlore-type Y_2_[Mn_x_Ru_1−x_]_2_O_7_ electrocatalyst

The mixed B-site pyrochlore-type catalyst Y_2_[Mn_x_Ru_1−x_]_2_O_7_ was prepared using a sol-gel method [20]. In a typical experiment, 1 mmol of Y(NO_3_)_3_·6H_2_O (0.3830 g) was mixed with stoichiometric amount of Ru(NO)(NO_3_)_x_(OH)_y_ and Mn(NO_3_)_2_·4H_2_O with a total molar amount of 1 mmol in 10 mL of water in a 50-mL beaker, followed by the addition of 4 mmol of citric acid (0.7685 g). The amount of precursors used in different samples was summarized in Additional file 1: Table S1. The beaker was transferred to an oil bath, heated to 80 °C using magnetic stirrer/hot plate (VWR, Cat. No. 97042-714), and kept at this temperature for 5 h to allow water to evaporate and then placed in a vacuum oven (VWR Symphony, E191047) at 120 °C for additional 6 h to further remove the moisture. The obtained solid was grinded with a pestle and mortar for a uniform dispersion, and then transferred into alumina boat (Sigma Aldrich, 5 mL). This combustion boat was placed in a tube furnace (Thermo Fisher Scientific™, Lindberg/Blue M™ Mini-Mite™) and heated up to 600 °C at a ramping rate of 5 °C/min and maintained at this temperature for 6 h. After being cooled down to room temperature, the solid was grinded again and heated to 1000 °C at a rate of 5 °C/min and maintained at this temperature for 12 h in the tube furnace.

### Synthesis of Ir-based pyrochlore-type electrocatalyst

The Ir-based oxide reference catalysts, Y_2_Ir_2_O_7_ and Y_2_[Mn_0.5_Ir_0.5_]_2_O_7_, were prepared by following the protocols described elsewhere [30]. Iridium chloride hydrate was used as the precursor for the preparation of both Ir-based reference catalysts.

### Synthesis of Y_2_Mn_2_O_7_ electrocatalyst

Pyrochlore Y_2_Mn_2_O_7_ was prepared using an assisted metathesis method [52]. In general, MnO_2_ (1 mmol or 0.0869 g), YCl_3_·6H_2_O (1 mmol or 0.3034 g) and Na_2_CO_3_·H_2_O (1.5 mmol or 0.1860 mg) was mixed with a pestle and mortar until a uniform mixture was obtained. The powder was then heated in the tube furnace in O_2_ to 650 °C with a ramping rate of 5 °C/min and maintained at this temperature for 24 h.

### Characterizations

X-ray diffraction (XRD) data was measured between 10° and 80° 2θ at a scan rate of 0.04° 2θ per second using a Rigaku Miniflex 600 diffractometer with Cu Kα X-ray source (λ = 1.54056 Å). The powder sample was evenly spread in the sample holder. High-resolution transmission electron microscopy (HR-TEM) images were obtained using JEOL 2100 Cryo TEM with a LaB_6_ emitter at an acceleration voltage of 200 kV. TEM samples were prepared by depositing a drop of dispersion of catalysts in ethanol on carbon coated TEM grids. Scanning electron microscopy (SEM) images were collected using a Hitachi S4800 microscope at an acceleration voltage of 10 kV. The specimen was prepared by placing powder samples onto the sample holder using carbon tape. Energy dispersive X-ray fluorescence (EDXRF) was performed on a Shimadzu EDX-700 spectrometer with Rh X-ray source. The powder samples were added to a polypropylene sample cup with an ultralene film at the bottom for testing. X-ray photoelectron spectroscopy (XPS) analysis was performed using a Kratos Axis ULTRA with an Al Kα X-ray source. The data processing and peak fitting were performed using the CasaXPS software.

### Electrochemical measurements

A standard three-electrode system was used in the study of electrochemical activity with a CHI 760E potentiostat (CH Instruments, Inc.). A platinum wire connected with platinum foil was used as the counter and a reversible hydrogen electrode (RHE, Hydroflex) was used as the reference electrode. A catalyst-loaded rotating disk electrode (RDE) with an area of 0.196 cm^2^ was used as the working electrode. Perchloric acid (0.1 M) was used as the electrolyte. The system was calibrated in H_2_-saturated 0.1 M HClO_4_ solution before test. All measurements were performed after purging the solution with O_2_ for at least 30 min. To prepare ink, 2 mg of catalysts, 2 mg of carbon black (XC-72), and 3 µL of neutralized Nafion solution were added in 2 mL of THF in a vial. The neutralized Nafion solution (pH = 7) was prepared using Nafion 117 aqueous solution mixed with 0.1-M NaOH solution. The vial was then put in an ice bath to sonication for 30 min to form a uniform dispersion. To prepare the catalyst-loaded working electrode, 5 µL of the prepared ink was drop-cast onto a rotating disk electrode (RDE) using a pipette, followed by dropping 5 µL of a Nafion-THF solution (3 µL neutralized Nafion solution in 2 mL of THF) twice onto the RDE. The ink was allowed to dry slowly to form a thin film working electrode under ambient conditions.

The cyclic voltammogram (CV) curves were collected at a scan rate of 10 mV/s, typically between 1.1 and 1.7 V vs. RHE for at least 5 cycles, the third CV scan was used for the analysis. The average of the anodic and cathodic current of CV data was finally exhibited as the current of the polarization curve in this paper to analyze the OER activity. The RDE rotating speed was set at 1600 rpm.

The double layer capacitance (C_dl_) was measured by conducting CV scans at scan rates of 10, 20, 30, 40 and 50 mV/s, respectively, in a non-Faradaic potential range from 1.1 to 1.2 V vs. RHE. The differences between anodic and cathodic current density at the potential of 1.15 V was divided by two and plotted versus scan rates. The C_dl_ values of the samples were calculated from the slope.

For stability, chronopotentiometry tests were conducted under the current density of 10 mA/cm^2^ using carbon paper as the substrate. Seven hundred fifty microliters of ink was deposited on the carbon paper (FuelCellStore, Sigracet 22 BB) with a working area of 1 × 1 cm^2^.

The turnover frequency (TOF) was estimated based on the measured current density using the following formula:

$${\text{TOF}} = \frac{{{\text{number}}\;{\text{of}}\;{\text{generated}}\;{\text{O}}\;{\text{molecules}}}}{{{\text{number}}\;{\text{of}}\;{\text{Ru}}\;{\text{cations}}}}$$

where the number of generated O molecules is obtained from the measured current density, assuming 100% Faradaic efficiency [20].

## Results and discussion

**Fig. 1:**
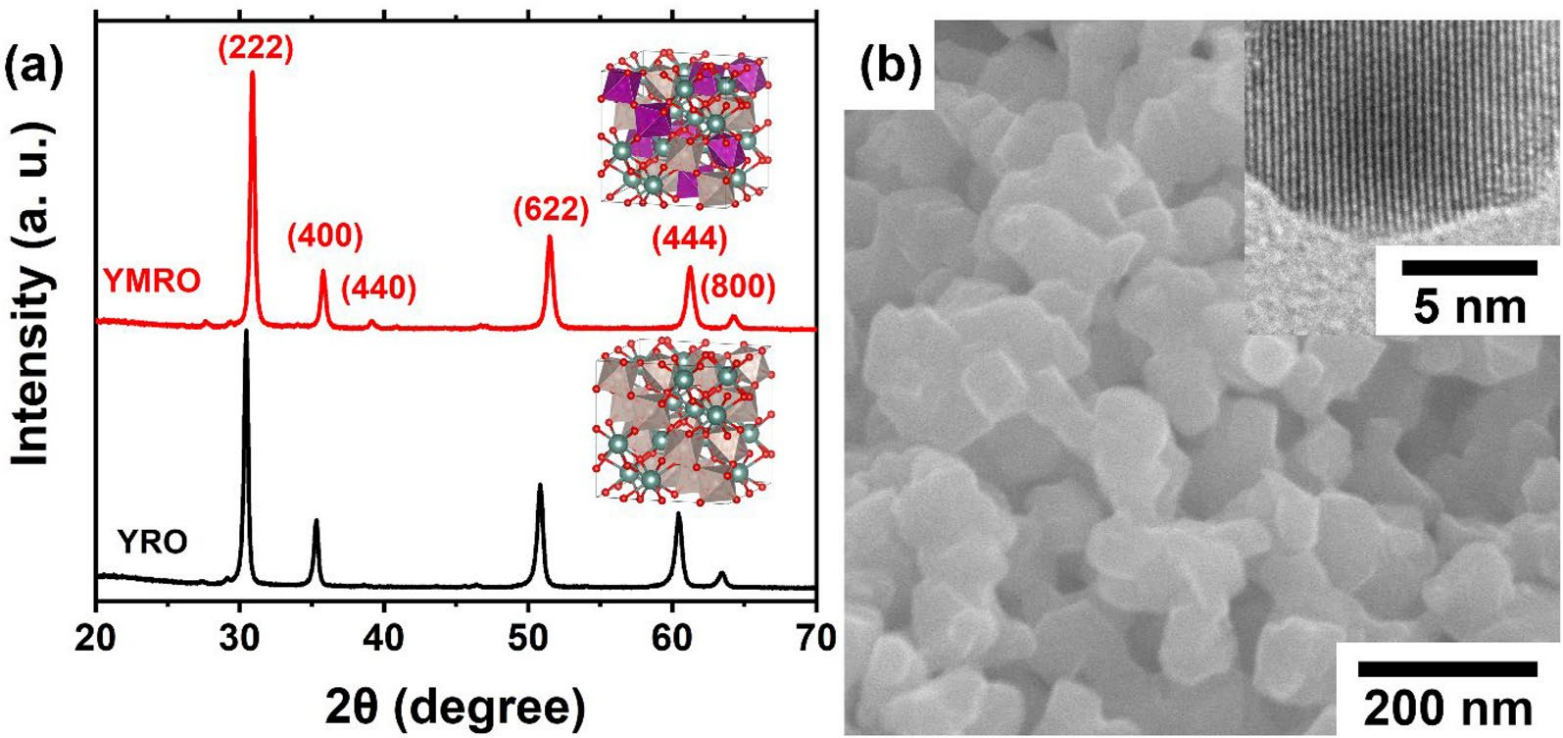
**a** XRD patterns and the coresponding illustrations of crystal structure (inset), **b** SEM and TEM (inset) images of YMRO. Color code: Y (teal), Ru (gray), Mn (purple), and O (red). The XRD pattern for YRO was inlcuded for comparison

Phase pure Y_2_[Mn_0.5_Ru_0.5_]_2_O_7_ (YMRO) was prepared using a modified method reported previously [20]. Figure 1a shows the XRD pattern of the YMRO compound. All diffraction peaks can be assigned to the cubic phase pyrochlore in Fd-3m space group and no impurity phase was detected. In comparison with the XRD pattern of pyrochlore Y_2_Ru_2_O_7_ (YRO, PDF#: 01-81-2340), all of the peaks shift to higher angles, indicating the metal substitution on the B-site. The inset of Fig. 1a illustrates the crystal structure of YMRO. Ruthenium and Mn should randomly distribute on the B-site of pyrochlore, each connects with neighbor O atoms to form RuO_6_ or MnO_6_ octahedrons, and RuO_6_ octahedrons are usually the active structures for OER. SEM image shows that the powder samples of YMRO were made of ~ 50-nm-sized primary particles (Fig. 1b). The TEM image shows that YMRO particles were highly crystalline. The *d*-spacing was determined to be 3.0 Å, which corresponds to the lattice spacing of (222) plane of pyrochlore. The bright spots in the selected area electron diffraction (SAED) pattern further suggest the high crystallinity of as-prepared YMRO (Additional file 1: Figure S1).

**Fig. 2:**
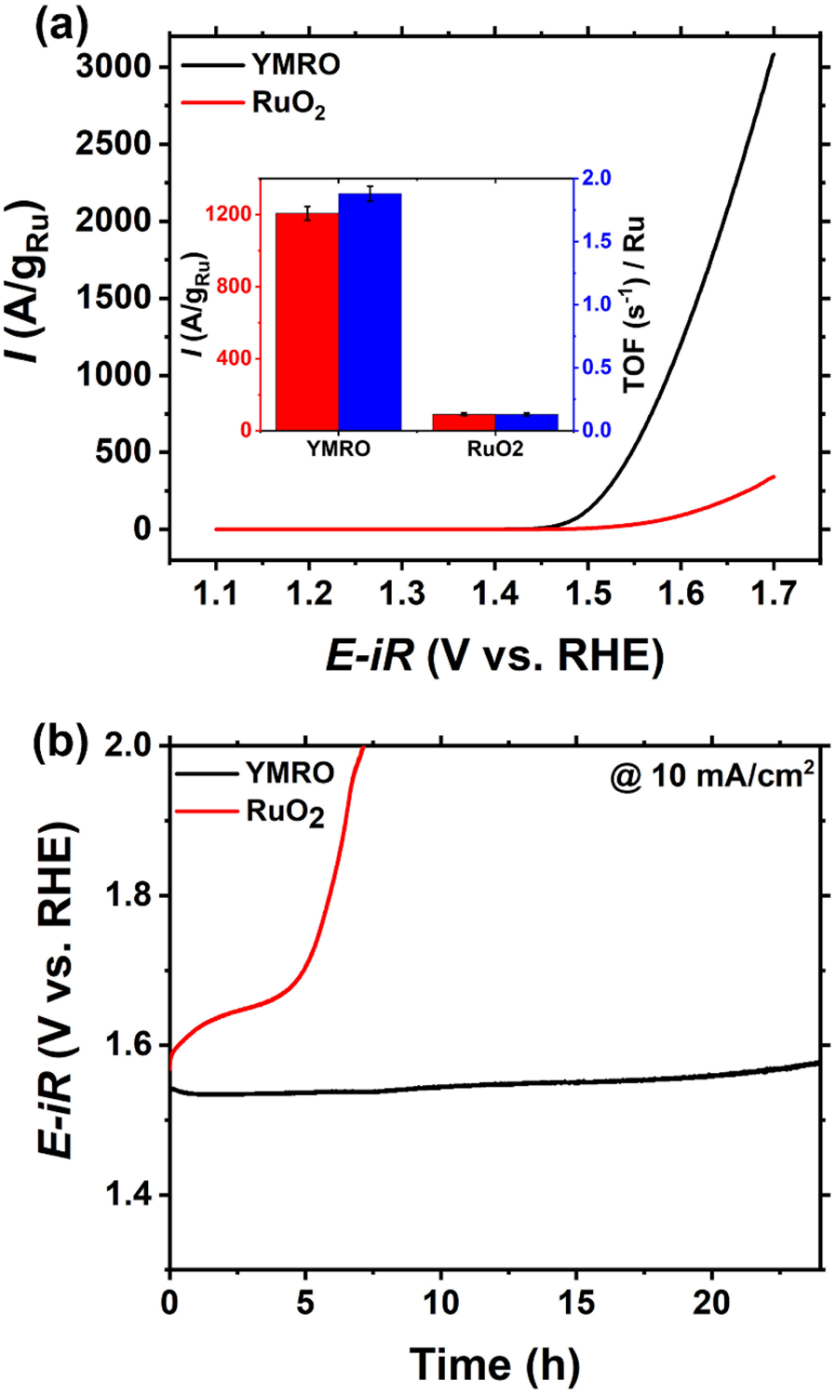
OER performance of pyrochlore YMRO and reference RuO_2_ electrocatalysts. **a** Polarization curves, the inset shows the comparison of current densities and TOF at 1.60 V versus RHE for YMRO and RuO_2_. **b** Chronopotentiometry performance of YMRO and RuO_2_ under constant current density of 10 mA/cm^2^ electrode up to 24 h

The OER performance was analyzed in 0.1-M HClO_4_ electrolyte using a three-electrode system. Figure 2a shows the polarization curve of YMRO electrocatalyst, and the comparison of its performance with a reference RuO_2_ catalyst. The working potential of YMRO at the current density of 5 mA/cm^2^ was measured to be ~ 1.57 V vs. RHE, corresponding to an overpotential (η) of ~ 340 mV, which is lower than that of RuO_2_ (~ 1.68 V vs. RHE, η ≈ 450 mV) (Additional file 1: Figure S2). The bar graph, shown in the inset of Fig. 2a, summarizes the comparison of OER performance between YMRO and RuO_2_ electrocatalysts. At the potential of 1.60 V, YMRO exhibits a current density of 1207 A/g_Ru_, which is over 13 times higher than that of RuO_2_ (92 A/g_Ru_). The YMRO electrocatalyst exhibited a high TOF of 1.88 s^−1^ at 1.60 V, while it is 0.13 s^−1^ for RuO_2_ measured under the same potential .Since RuO_2_ and YMRO had different particle sizes (Additional file 1: Figure S3), double layer capacitance (C_dl_) for these two electrocatalysts were characterized to estimate the electrochemically active surface area (ECSA) to further compare and understand their intrinsic activity. Additional file 1: Figure S4a–c show the CV scans of YMRO and RuO_2_ at different scan rates in a non-Faradaic potential range between 1.1 and 1.2 V vs. RHE and their corresponding linear fitting. Our data show RuO_2_ exhibited a higher C_dl_ of 5.32 mF/cm^2^ than that for YMRO, which is 3.44 mF/cm^2^, indicating that the RuO_2_ reference catalyst actually has a larger ECSA. Additional file 1: Figure S4d shows the C_dl_-normalized OER activity values for YMRO and RuO_2_, respectively. The activity of YMRO is 2.31 mA/mF at the potential of 1.60 V, which is seven times higher than that of RuO_2_ (0.33 mA/mF). This result indicates YMRO catalysts have higher intrinsic OER activity than RuO_2_; in another word, specific surface area should not play the major role in the observed, enhanced activity. The stability of YMRO and RuO_2_ in acidic media was examined based on chronopotentiometry at the constant current density of 10 mA/cm^2^. The overpotential was around 300 mV for YMRO electrocatalyst (Fig. 2b), and this value remained constant for the measuring time period of 24 h, demonstrating a good stability. In comparison, the potential of RuO_2_ started from ~ 1.58 V and gradually increased to ~ 1.68 V in 5 h, and then rapidly increased to well above 2 V in 7 h, indicating the RuO_2_ reference catalyst quickly lost its activity during this testing period.

**Fig. 3:**
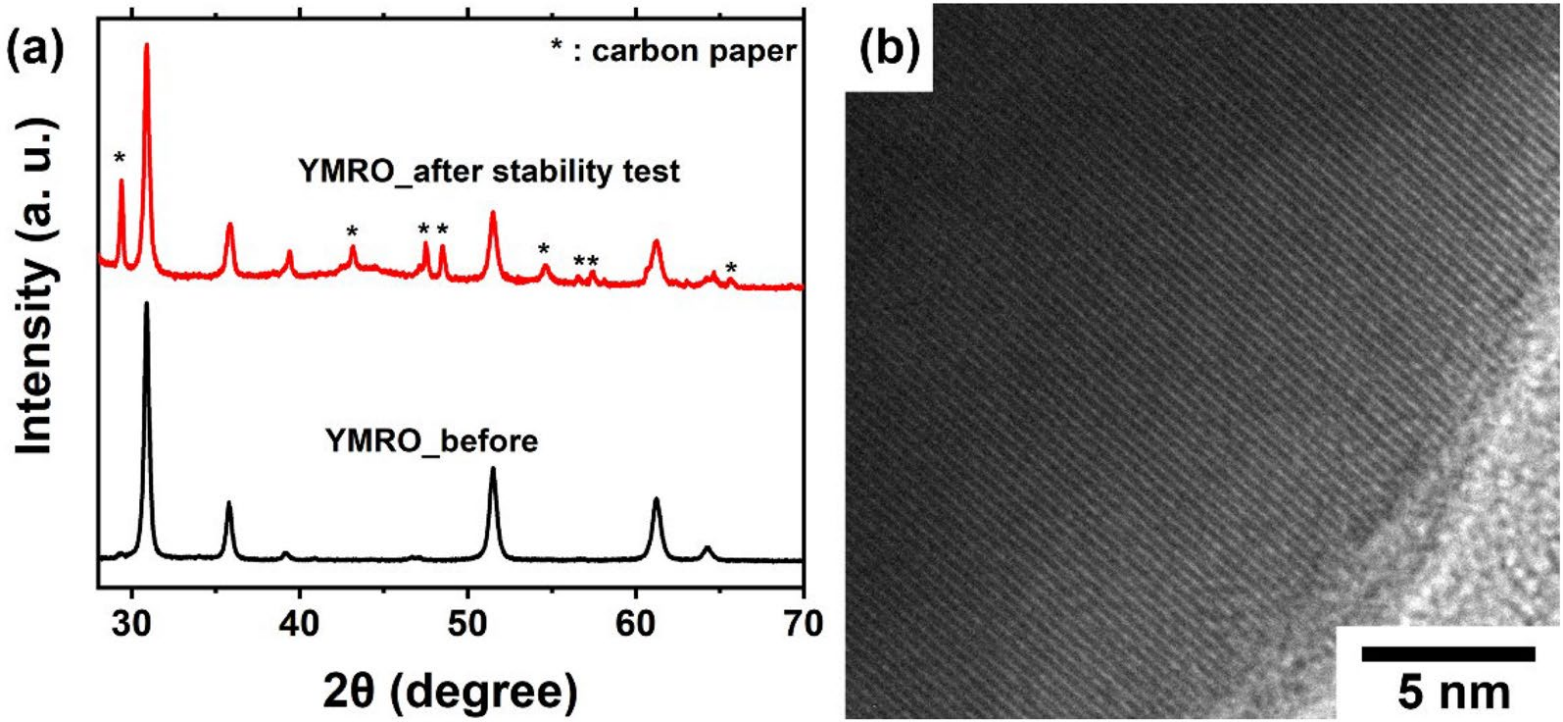
**a** XRD pattern and **b** TEM image of YMRO after the stability tests

XRD and TEM were applied to study the crystal structures of the YMRO electrocatalysts after the stability tests in acid. Figure 3a shows the XRD patterns of the YMRO electrocatalyst before and after the test. There is no visible difference between the XRD patterns of the YMRO pyrochlore. The extra peaks for the sample after the stability study were all from carbon paper used as the support for electrocatalysts in the electrode. The structural stability was further examined by TEM on the samples after the OER stability test. There was no obvious change in the lattice or the formation of amorphous structure when compared with that of the as-made YMRO (Figs. 1b and 3b). In comparison, clear surface structural change was observed for RuO_2_ after the OER tests [20, 40].

**Fig. 4:**
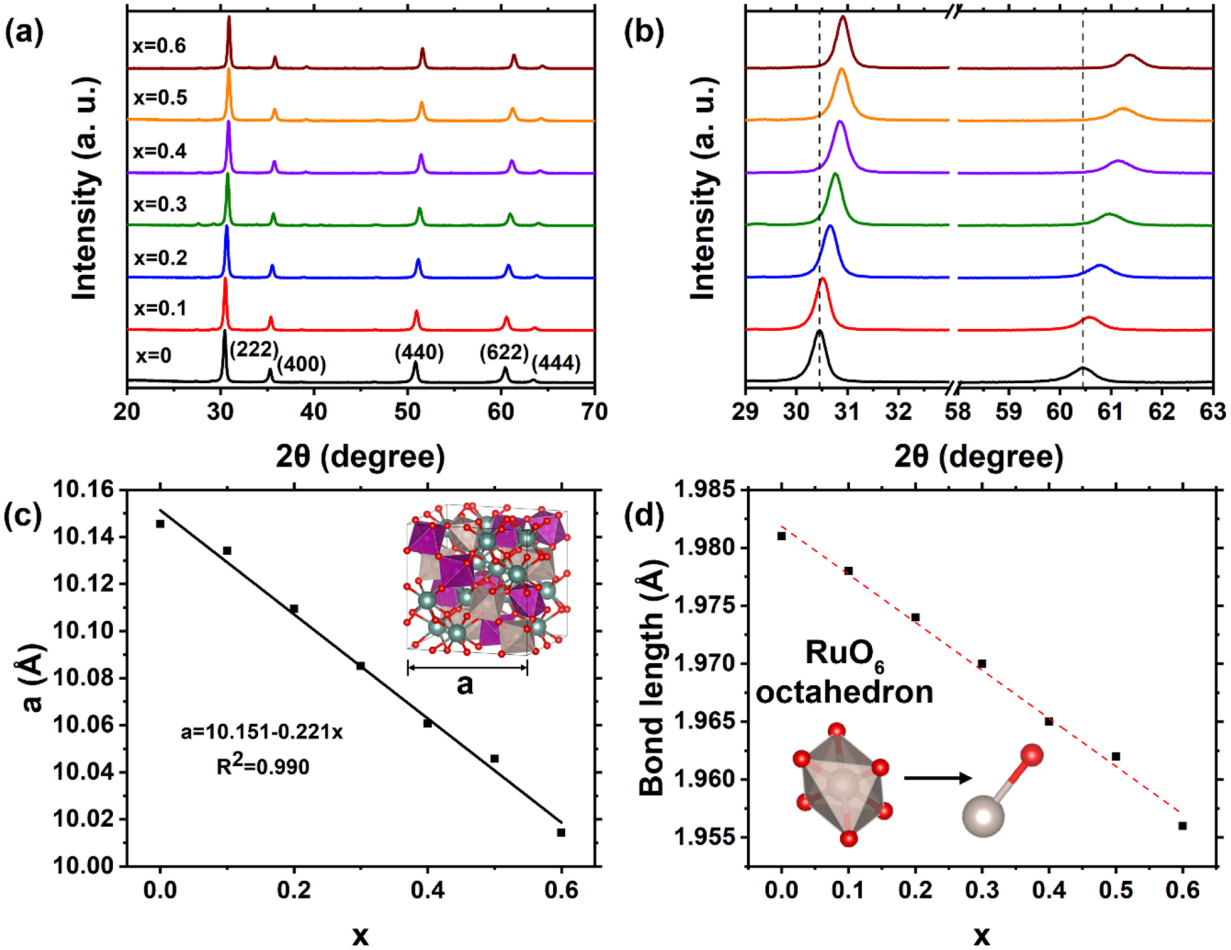
**a**, **b** XRD patterns, **c** lattice parameter and **d** Ru-O bond length of YMRO-x (x = 0-0.6) electrocatalysts

A series of Y_2_[Mn_x_Ru_1−x_]_2_O_7_ pyrochlore with different B-site metal ratios (denoted as YMRO-x) were prepared to further study the effects of metal substitution on the OER performance (Additional file 1: Table S1). Figure 4a and b show the XRD patterns of YMRO-x (x = 0-0.6). All diffraction peaks of YMRO-x shifted to high angles compared with YRO (PDF#: 01-81-2340), indicating that the length of unit cells was reduced based on the Bragg’s law calculation. As more Ru ions were substituted by Mn cation in these pyrochlore structures, the peak monotonically shifted to higher angles. For YMRO-0.5, the peak shifted by 0.44° 2θ for the (222) diffraction plane and 0.80° 2θ for the (622) plane. The lattice parameter of cubic pyrochlore and Ru-O bond length in RuO_6_ octahedrons was analyzed by Rietveld refinement with XRD data. Figure 4c shows the lattice parameters of pyrochlore decreases linearly as Mn replaces Ru, this reduced size of unit cell is attributed to the smaller ionic radius of Mn^4+^ cation compared with Ru^4+^. Figure 4d shows the change of Ru-O bond length in RuO_6_ octahedrons, which decreases with the increase of x value. The shortened Ru-O bond is often the result of increased overlap between Ru 4d and O 2p orbitals, which facilitates the kinetics of OER [36]. Additional file 1: Figure S5 shows the SEM images of these YMRO-x oxides. The overall morphology of all the samples were similar to that of YMRO-0.5 in Fig. 1b. The atomic ratios of metal elements were obtained by EDXRF and consistent with the feeding ratios of the metal precursors (Additional file 1: Table S2). It appears the maximum of x value is around 0.6 for this series of YMRO-x oxides. When x value was 0.7 or larger, YMRO-x could not be prepared in a pure pyrochlore phase using the method developed (Additional file 1: Figure S6). When x was equal to 0.7 (i.e., nominal formula of Y_2_[Mn_0.7_Ru_0.3_]_2_O_7_), both pyrochlore phase and hexagonal phase YMnO_3_ (PDF#: 00-025-1079) were detected. In this study, the reference pyrochlore Y_2_Mn_2_O_7_ (YMO) was synthesized through an assisted metathesis method as a control sample [52]. X-ray diffraction study indicates that YMO was formed, together with small amount of the Y_2_O_3_ impurity in the sample (Additional file 1: Figure S7).

**Fig. 5:**
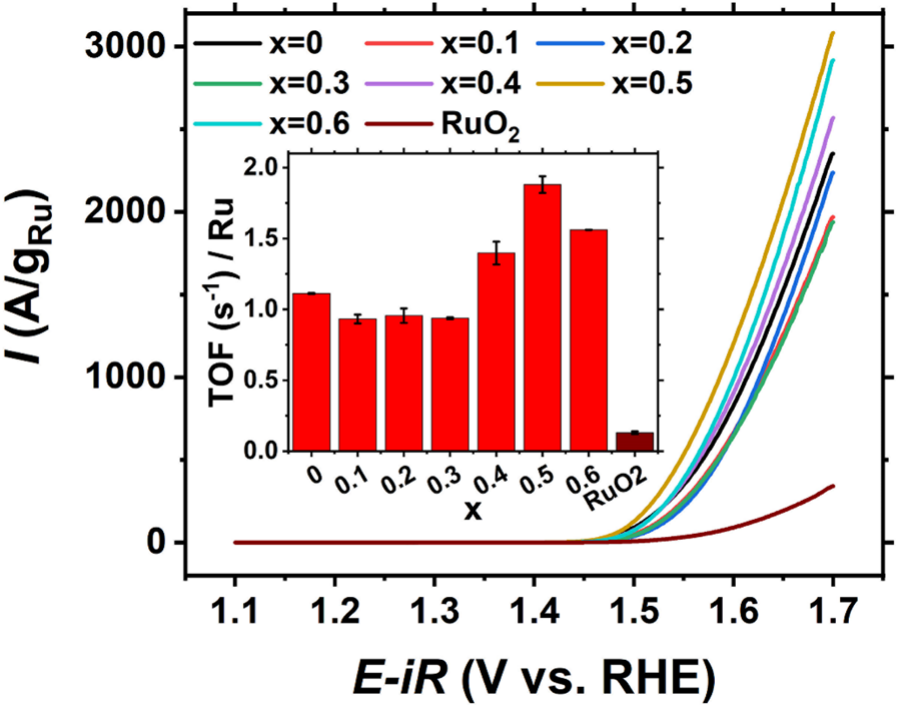
Polarization curves of YMRO-x and reference RuO_2_ electrocatalysts, the inset shows the comparison of TOF at 1.60 V versus RHE for YMRO-x and RuO_2_

Figure 5 shows the OER performance of this series of YMRO-x. Among these catalysts, YMRO-0.5 exhibits the highest current density at the potential of 1.70 V, which is 3082 A/g_Ru_. The OER specific activity of all the YMRO-x samples is higher than 1900 A/g_Ru_, while YMRO-0.1, 0.2 and 0.3 show the lowest activity among the series. In comparison, the current density of RuO_2_ is only 342 A/g_Ru_ at 1.70 V. The inset of Fig. 5 shows the calculated TOF per unit Ru atom for these YMRO-x catalysts. While YMRO-0, 0.1, 0.2 and 0.3 show similar TOF of around 1.0 s^-1^, YMRO-0.5 exhibits the highest TOF of 1.88 s^−1^. When the x value further increased to 0.6, TOF decreased to around 1.6 s^−1^. All YMRO-x samples showed higher TOF values than that of the reference RuO_2_, which was determined to be 0.13 s^−1^. Additional file 1: Figure S8 shows the geometric activity of YMRO-x, where YRO exhibits highest geometric current density among the series due to its highest Ru ratio. This result differs from a previous report, in which Y_2_Mn_0.1_Ru_1.9_O_7_ outperformed YRO [41]. This apparent difference can be explained by the tradeoff between the Ru amount in the catalyst and the mass activity of Ru. Manganese substitution on the B-site enhanced the mass activity per Ru. A higher level of substitution, which reduces the Ru amount in the catalysts, however leads to a reduced geometric activity. To determine the contribution of B site cations (Ru and Mn) to the overall OER performance in YMRO, we ran the polarization curve of the Y_2_Mn_2_O_7_ sample (Additional file 1: Figure S9). The result shows Y_2_Mn_2_O_7_ exhibited negligible activity, indicating that Ru cation is the catalytic center.

**Fig. 6:**
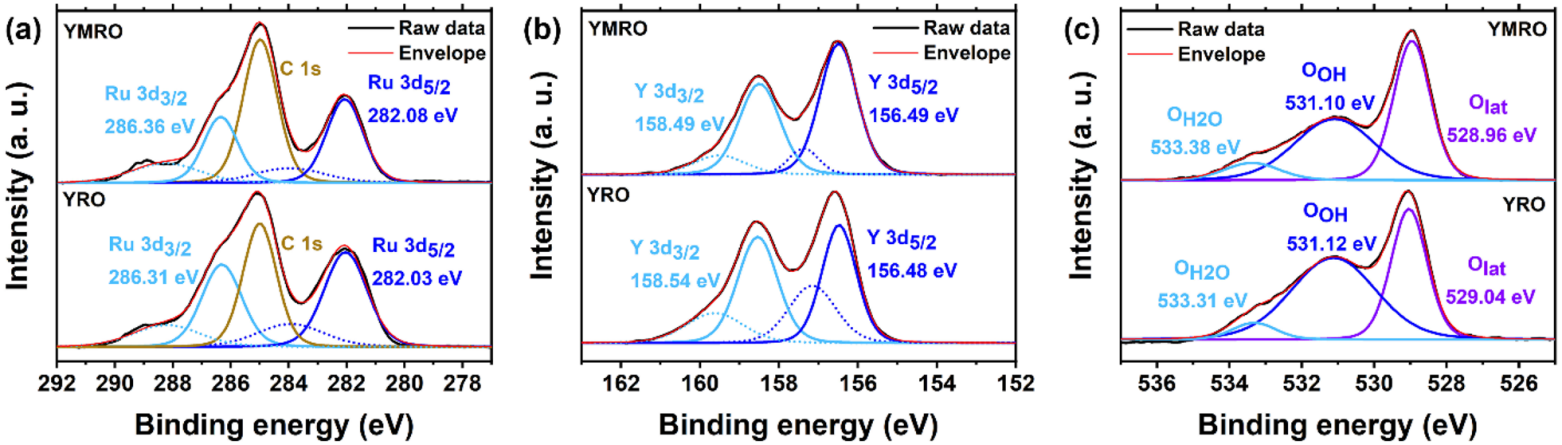
XPS spectroscopy of the **a** Ru 3d, **b** Y 3d and **c** O 1 s regions of YMRO and YRO samples

XPS was conducted to analyze the surface properties to understand the observed TOF trends of this series of YMRO electrocatalysts. Additional file 1: Figure S10 shows the XPS survey scans of YMRO and YRO reference, respectively. The main difference between the two is the Mn 2p peaks at ~ 640 eV. Figure 6a and Additional file 1: Table S3 shows the XPS of the Ru 3d region of YMRO and YRO. The spectra could be fitted into one C 1 s peak, two Ru 3d peaks and their corresponding satellite peaks. In comparison to YRO, the Ru 3d_3/2_ and Ru 3d_5/2_ peaks slightly shifted to higher binding energy, indicating that the oxidation state of Ru increased after the B-site substitution with Mn cations. Transition of Ru(IV) to a higher oxidation state is often critical in the potential determining step (PDS) in the OER pathways and could facilitate the fast OER kinetics [36]. The XPS spectra of Y 3d region shows that the YMRO has an energy gap of 0.06 eV between *E*_*Y3d3/2*_ and *E*_*Y3d5/2*_, which is smaller than that for YMO (Fig. 6b and Additional file 1: Table S4). The smaller energy gap between *E*_*Y3d3/2*_ and *E*_*Y3d5/2*_ suggest it is easier to reduce Y(III) by the oxidation of Ru(IV), favoring a high OER kinetics [53].

The XPS of O 1 s spectra were fitted into three species, namely, lattice oxygen (O_lat_), hydroxyl group (O_OH_) and adsorbed water (${\text{O}}_{{\text{H}}_{2} {\text{O}}}$) (Fig. 6c and Additional file 1: Table S5) [35]. The ratio between the area of O_OH_ and O_lat_ could be an indication of the concentration of hydroxyl species on the surface of the catalysts. A high hydroxyl level on the surface is usually beneficial to the OER activity [3, 47, 48]. For YMRO, the area percentage is 46.9% for O_lat_ and 44.4% for O_OH_ with an O_OH_/O_lat_ ratio of 0.95. In comparison, YRO has a much higher O_OH_/O_lat_ ratio of 1.47, indicating a higher density of hydroxyl group on the surface of YRO. Our data suggest that the oxidation state of Ru is more critical in regulating the TOF of these YMRO electrocatalysts than the surface hydroxyl groups, since YMRO exhibited higher TOF than YRO.

**Fig. 7:**
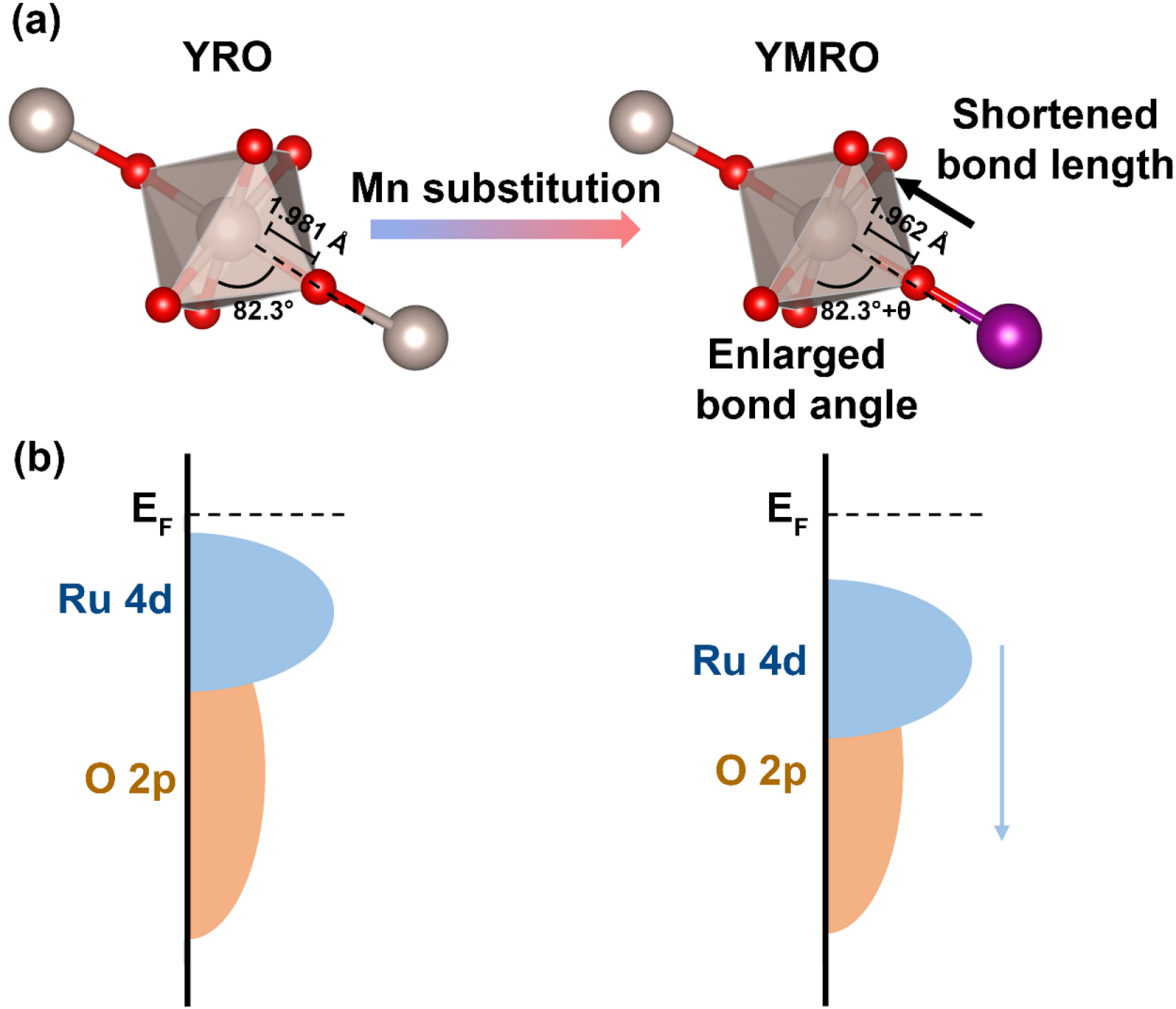
Illustration of **a** the normal and distorted RuO_6_ building blocks and **b** the corresponding shifts of band center of O 2p and Ru 4d bands

The TOF trend in this series of YMRO electrocatalysts could be further explained from the electronic structure. As illustrated in Fig. 7a, RuO_6_ exists in an octahedral symmetry in YRO, with the Ru-O bond length of 1.981 Å and O-Ru-O bond angle of 82.3°. Upon the replacement of Ru by Mn, the length of the neighboring Ru-O bond was shortened to 1.962 Å due to the higher absolute electronegativity of Ru^4+^ than that of Mn^4+^. The change in electric field resulted from the introducing of Mn^4+^ could lead to the distortion of RuO_6_ octahedron and an increased O-Ru-O angle. The shortened Ru-O bond length and the enlarged O-Ru-O bond angle result in an increased overlap between O 2p and Ru 4d orbitals, as well as weakened Jahn-Teller distortion, leading to a broadened Ru 4d band width and lowered band center, which is beneficial to the OER kinetics (Fig. 7b) [36].

**Fig. 8:**
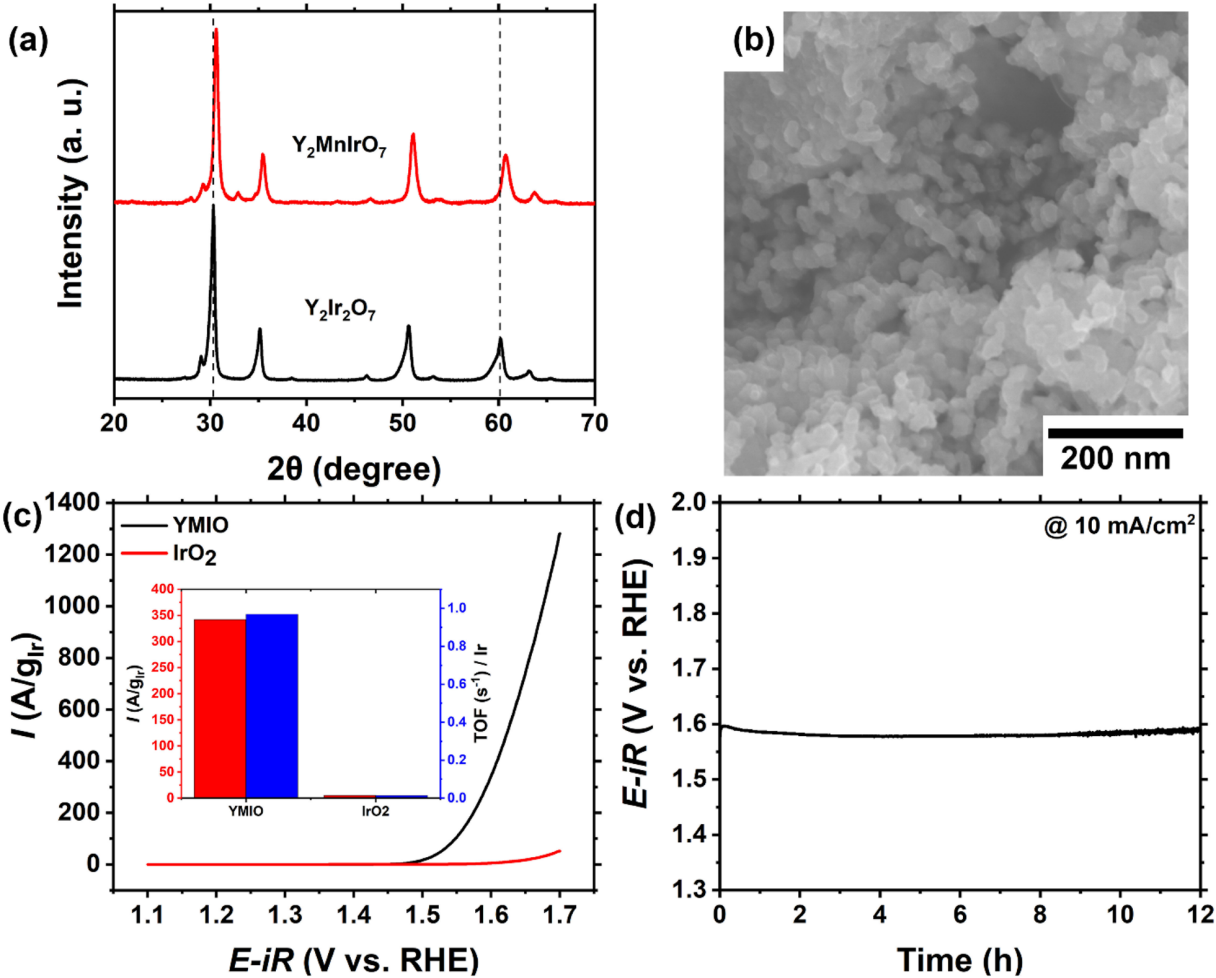
**a** XRD pattern, **b** SEM image of YMIO, **c** OER activity of YMIO and IrO_2_, the inset shows the comparison of current densities and TOF at 1.60 V versus RHE for YMIO and IrO_2_. **d** Chronopotentiometry of YMIO under constant current density of 10 mA/cm^2^ electrode up to 12 h

The strategy for increasing the OER activity through controlling the mixing of B-site cations should in principle be applicable to other pyrochlore-type electrocatalysts. Thus, we prepared pyrochlore Y_2_MnIrO_7_ (YMIO) using the same general method and examined its OER performance. The XRD patterns show the diffraction peaks of YMIO shift to higher angle than those of Y_2_Ir_2_O_7_ because of the reduction of the unit cell due to the replacement of Ir by Mn cations (Fig. 8a). SEM image shows the submicron-sized particle of YMIO (Fig. 8b). Figure 8c shows the OER activity of YMIO electrocatalyst in comparison with a IrO_2_ reference catalyst (Fig. 8c). The current density of YMIO was determined to be 342 A/g_Ir_ at the potential of 1.60 V, which is over 68 times higher than that of IrO_2_ (5 A/g_Ir_). The TOF per Ir atom was measured to be 0.97, which is around 75 times higher than that for IrO_2_ (0.013 s^-1^). The overpotential of YMIO was determined to be around 390 mV based on the chronopotentiometry of YMIO at the constant current density of 10 mA/cm^2^. This value maintained well over the testing period of 12 h in 0.1 M HClO_4_ electrolyte.

## Conclusions

In summary, we prepared a series of nm-scaled, mixed B-site pyrochlore Y_2_[Mn_x_Ru_1-x_]_2_O_7_ electrocatalysts. Among these samples, Y_2_[Mn_0.5_Ru_0.5_]_2_O_7_ exhibits the highest TOF, which is around 1.7 times higher than Y_2_Ru_2_O_7_ and 14 times higher than the RuO_2_ reference catalyst. Oxidation state of Ru appears to play an essential role (vs. surface density of hydroxyl group) in regulating OER performance in the mixed B-site pyrochlore OER catalysts. Change of electronic structure due to B-site substitution by a 3d transition metal (Mn) cation favors the formation of broadened Ru 4d band width and lowered band center, resulting in the enhanced TOF of OER electrocatalysts. This B-site substitution strategy works for both Ru- and Ir-based pyrochlore (Y_2_MnIrO_7_ and Y_2_MnRuO_7_) OER catalysts. Thus, the approach presents a general strategy for enhancing the OER performance of electrocatalysts of complex oxides.

## Supplementary Information

**Additional file 1: Figure S1.** SAED pattern of Y_2_MnRuO_7_ (YMRO). **Figure S2**. Geometric activity of Y_2_MnRuO_7_ (YMRO) and RuO_2_ electrocatalysts. **Figure S3**. Representative SEM image of the reference RuO_2_ electrocatalyst. **Figure S4**. Electrochemical properties of Y_2_MnRuO_7_ (YMRO) and RuO_2_ electrocatalysts: (a, b) CVs in a non-faradic current region (1.1-1.2 V vs. RHE) at scan rates of 10, 20, 30, 40 and 50 mV/s, respectively; (c) linear fitting curves of the capacitive current versus CV scan rate; and (d) intrinsic activity normalized by C_dl_. **Figure S5**. Representative SEM images of (a) Y_2_Ru_2_O_7_ (YRO), (b) Y_2_Mn_0.2_Ru_1.8_O_7_ (YMRO-0.1), (c) Y_2_Mn_0.4_Ru_1.6_O_7_ (YMRO-0.2), (d) Y_2_Mn_0.6_Ru_1.4_O_7_ (YMRO-0.3), (e) Y_2_Mn_0.8_Ru_1.2_O_7_ (YMRO-0.4) and (f) Y_2_Mn_1.2_Ru_0.8_O_7_ (YMRO-0.6) powders. **Figure S6**. Representative XRD patterns of Y_2_Mn_1.4_Ru_0.6_O_7_ (YMRO-0.7) and the references. **Figure S7**. Representative XRD patterns of Y_2_Mn_2_O_7_. **Figure S8**. Geometric activity of Y_2_[Mn_x_Ru_1-x_]_2_O_7_ (YMRO-x) and reference RuO_2_ electrocatalysts. **Figure S9**. Polarization curve of the Y_2_Mn_2_O_7_ electrocatalyst. **Figure S10**. XPS survey scans of (a) YMRO and (b) YRO electrocatalysts. **Table S1**. The amount of precursors used in the synthesis of Y_2_[Mn_x_Ru_1-x_]_2_O_7_. **Table S2**. XRF analysis of as-made YMRO-x. **Table S3**. XPS analysis of Ru 3d region of YMRO and YRO. **Table S4**. XPS analysis of Y 3d region of YMRO and YRO. **Table S5**. XPS analysis of O 1s region of YMRO and YRO.

## Data Availability

The datasets used and/or analysed during the current study are available from the corresponding author on reasonable request.
