# Supplementary material for: Regulating the electronic structures of mixed B-site pyrochlore to enhance the turnover frequency in water oxidation
Source: Nano Converg. 2022 May 18;9:22. doi: 10.1186/s40580-022-00311-z (PMC9117583; doi:10.1186/s40580-022-00311-z)
Supplement: Supplementary file 1 — Additional file 1: Figure S1. SAED pattern of Y2MnRuO7 (YMRO). Figure S2. Geometric activity of Y2MnRuO7 (YMRO) and RuO2 electrocatalysts. Figure S3. Representative SEM image of the reference RuO2 electrocatalyst. Figure S4. Electrochemical properties of Y2MnRuO7 (YMRO) and RuO2 electrocatalysts: (a, b) CVs in a non-faradic current region (1.1-1.2 V vs. RHE) at scan rates of 10, 20, 30, 40 and 50 mV/s, respectively; (c) linear fitting curves of the capacitive current versus CV scan rate; and (d) intrinsic activity normalized by Cdl. Figure S5. Representative SEM images of (a) Y2Ru2O7 (YRO), (b) Y2Mn0.2Ru1.8O7 (YMRO-0.1), (c) Y2Mn0.4Ru1.6O7 (YMRO-0.2), (d) Y2Mn0.6Ru1.4O7 (YMRO-0.3), (e) Y2Mn0.8Ru1.2O7 (YMRO-0.4) and (f) Y2Mn1.2Ru0.8O7 (YMRO-0.6) powders. Figure S6. Representative XRD patterns of Y2Mn1.4Ru0.6O7 (YMRO-0.7) and the references. Figure S7. Representative XRD patterns of Y2Mn2O7. Figure S8. Geometric activity of Y2[MnxRu1-x]2O7 (YMRO-x) and reference RuO2 electrocatalysts. Figure S9. Polarization curve of the Y2Mn2O7 electrocatalyst. Figure S10. XPS survey scans of (a) YMRO and (b) YRO electrocatalysts. Table S1. The amount of precursors used in the synthesis of Y2[MnxRu1-x]2O7. Table S2. XRF analysis of as-made YMRO-x. Table S3. XPS analysis of Ru 3d region of YMRO and YRO. Table S4. XPS analysis of Y 3d region of YMRO and YRO. Table S5. XPS analysis of O 1s region of YMRO and YRO. [file 40580_2022_311_MOESM1_ESM.docx]

Additional Information

Regulating the Electronic Structures of Mixed B-site Pyrochlore to Enhance the Turnover Frequency in Water Oxidation

Cheng Zhang,^†^ Fangfang Wang,^†,‡^ Beichen Xiong,^†^ Hong Yang^†,*^

†Department of Chemical and Biomolecular Engineering, University of Illinois at Urbana-Champaign, 600 S. Mathews Avenue, Urbana, Illinois 61801, United States

‡School of Mechanical and Power Engineering, Zhengzhou University, 100 Science Avenue, Zhengzhou, Henan Province 450001, People's Republic of China

^z^ Corresponding author. E-mail: hy66@illinois.edu

**Additional Figures**


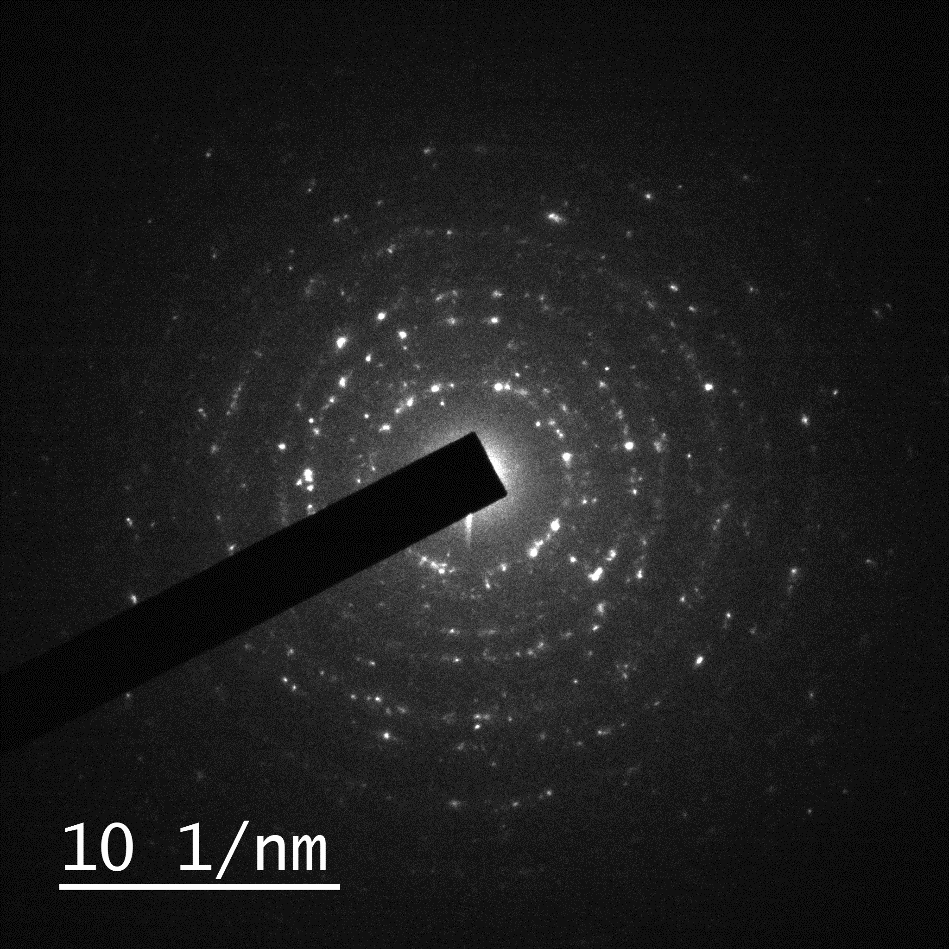


**Figure S1.** SAED pattern of Y_2_MnRuO_7_ (YMRO).





**Figure S2.** Geometric activity of Y_2_MnRuO_7_ (YMRO) and RuO_2_ electrocatalysts.


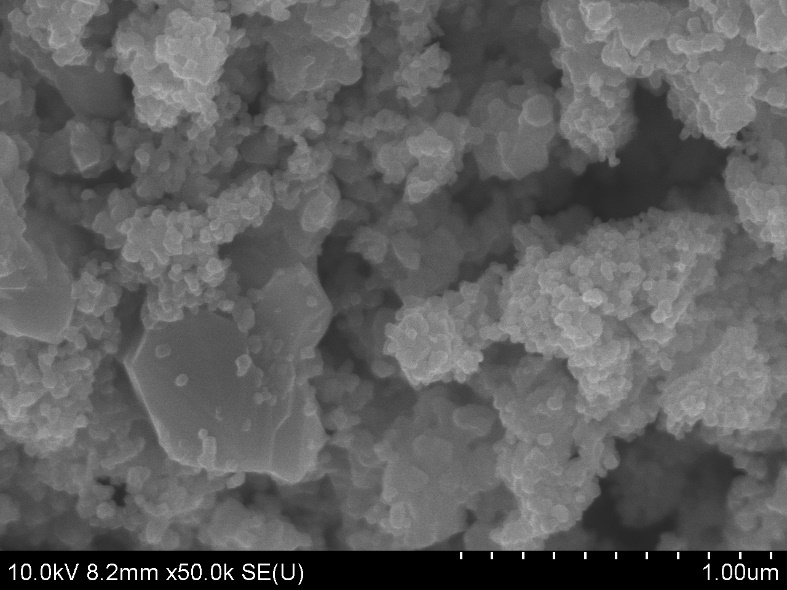


**Figure S3.** Representative SEM image of the reference RuO_2_ electrocatalyst.


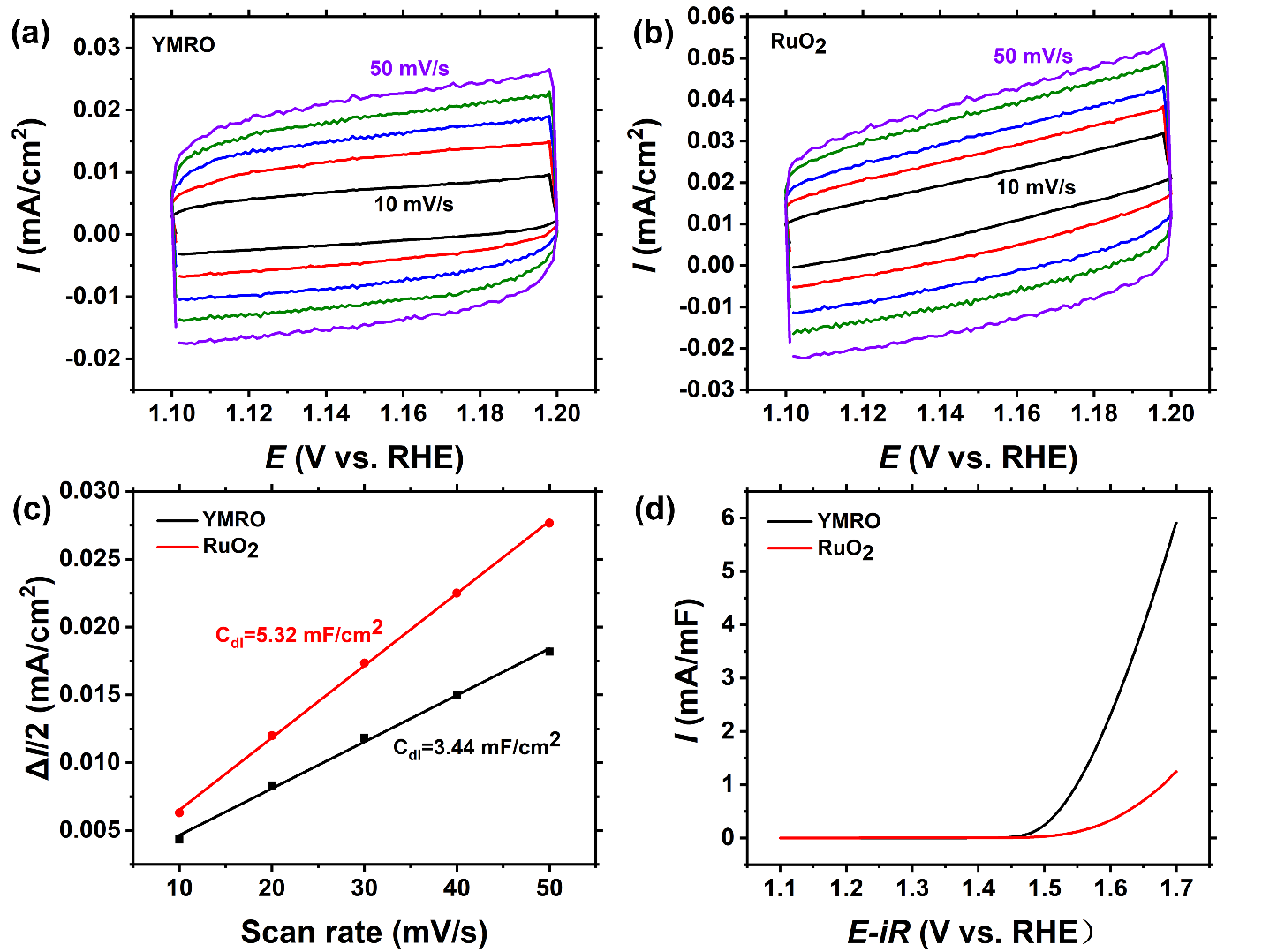


**Figure S4.** Electrochemical properties of Y_2_MnRuO_7_ (YMRO) and RuO_2_ electrocatalysts: (a, b) CVs in a non-faradic current region (1.1-1.2 V vs. RHE) at scan rates of 10, 20, 30, 40 and 50 mV/s, respectively; (c) linear fitting curves of the capacitive current versus CV scan rate; and (d) intrinsic activity normalized by C_dl_.

**
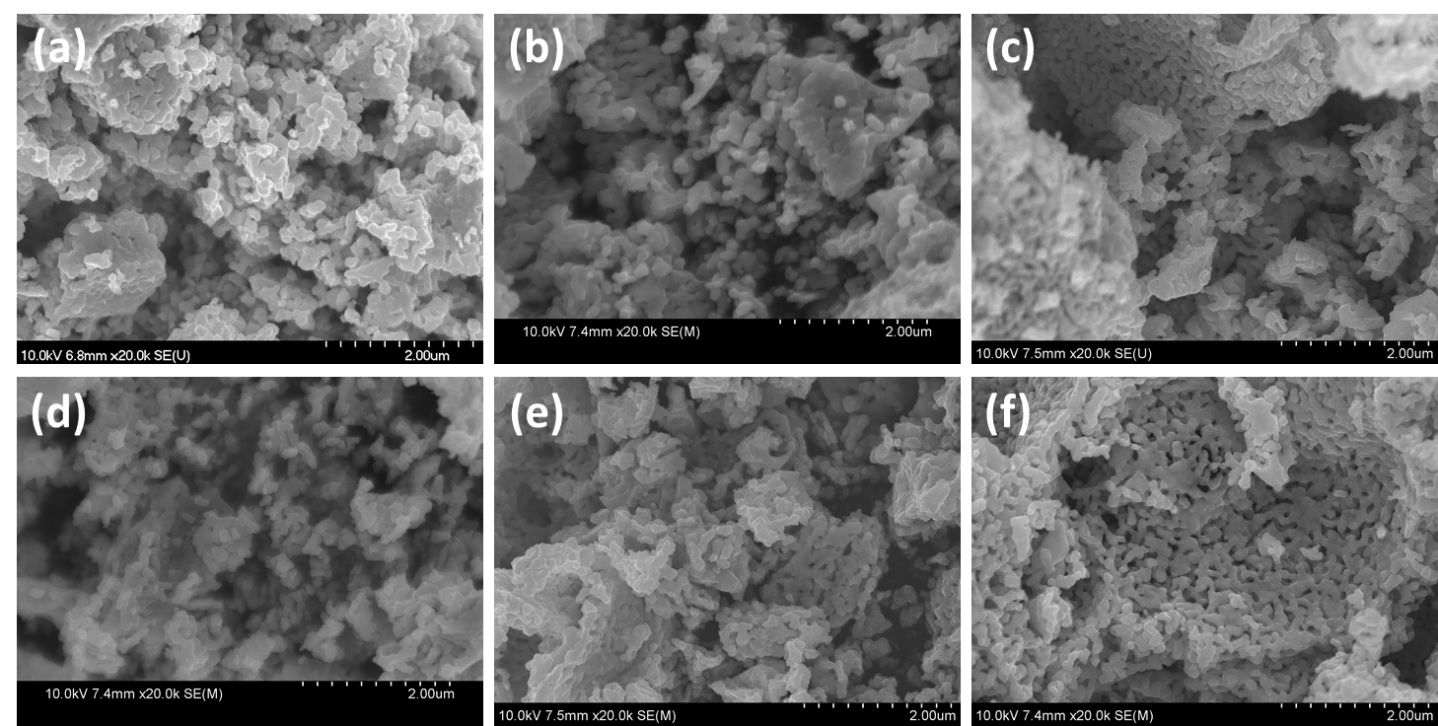
**

**Figure S5.** Representative SEM images of (a) Y_2_Ru_2_O_7_ (YRO), (b) Y_2_Mn_0.2_Ru_1.8_O_7_ (YMRO-0.1), (c) Y_2_Mn_0.4_Ru_1.6_O_7_ (YMRO-0.2), (d) Y_2_Mn_0.6_Ru_1.4_O_7_ (YMRO-0.3), (e) Y_2_Mn_0.8_Ru_1.2_O_7_ (YMRO-0.4) and (f) Y_2_Mn_1.2_Ru_0.8_O_7_ (YMRO-0.6) powders.





**Figure S6.** Representative XRD patterns of Y_2_Mn_1.4_Ru_0.6_O_7_ (YMRO-0.7) and the references.





**Figure S7.** Representative XRD patterns of Y_2_Mn_2_O_7_.





**Figure S8.** Geometric activity of Y_2_[Mn_x_Ru_1-x_]_2_O_7_ (YMRO-x) and reference RuO_2_ electrocatalysts.





**Figure S9.** Polarization curve of the Y_2_Mn_2_O_7_ electrocatalyst.


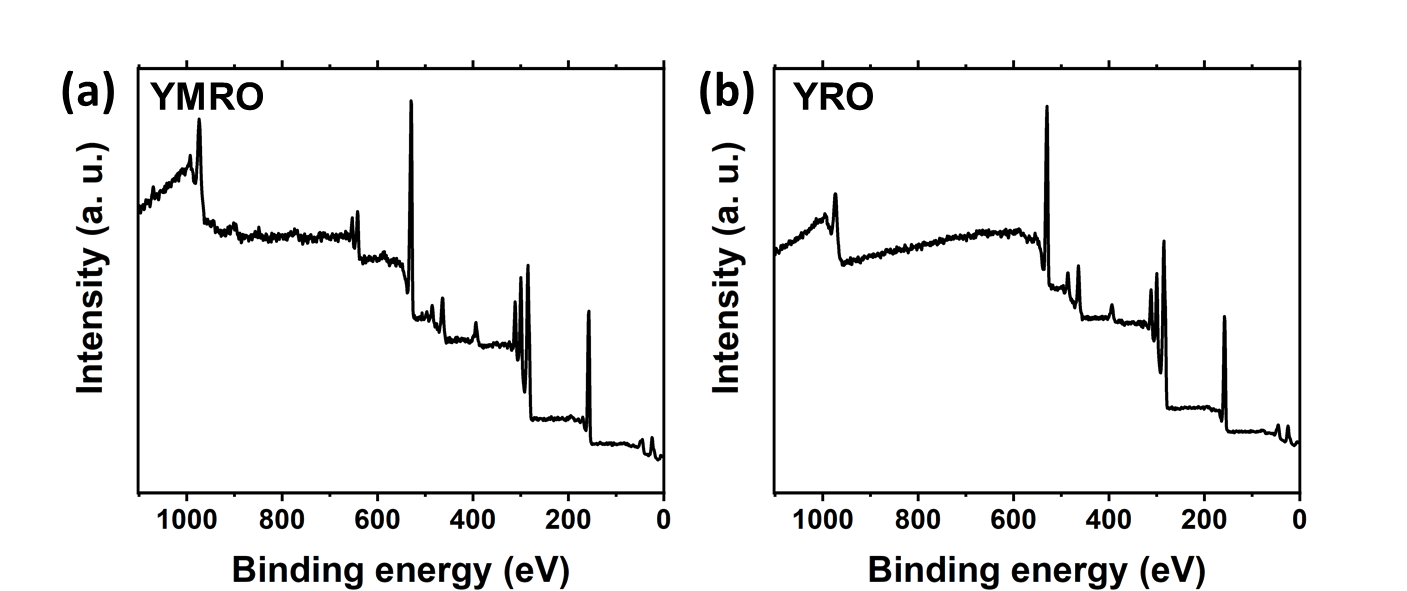


**Figure S10.** XPS survey scans of (a) YMRO and (b) YRO electrocatalysts.

**Tables**

**Table S1.** The amount of precursors used in the synthesis of Y_2_[Mn_x_Ru_1-x_]_2_O_7_.

| **x** | **Y(NO_3_)_3_·6H_2_O (g)** | **Ru(NO)(NO_3_)_x_(OH)_y_ (mL)** | **Mn(NO_3_)_2_·4H_2_O (g)** | **citric acid (g)** |
| --- | --- | --- | --- | --- |
| 0 | 0.3830 | 0 | 6.174 | 0.7685 |
| 0.1 | 0.3830 | 0.0251 | 5.557 | 0.7685 |
| 0.2 | 0.3830 | 0.0502 | 4.939 | 0.7685 |
| 0.3 | 0.3830 | 0.0753 | 4.322 | 0.7685 |
| 0.4 | 0.3830 | 0.1004 | 3.704 | 0.7685 |
| 0.5 | 0.3830 | 0.1255 | 3.087 | 0.7685 |
| 0.6 | 0.3830 | 0.1506 | 2.470 | 0.7685 |
| 0.7 | 0.3830 | 0.1757 | 1.852 | 0.7685 |

**Table S2.** XRF analysis of as-made YMRO-x.

| **x** | **Elemental ratio (atom %)** | | | **Mn:Ru ratio** |
| --- | --- | --- | --- | --- |
|  | **Y** | **Mn** | **Ru** |  |
| 0 | 60.255 | - | 39.745 | 0:1 |
| 0.1 | 58.345 | 4.926 | 36.728 | 0.12:0.88 |
| 0.2 | 64.465 | 10.574 | 24.960 | 0.30:0.70 |
| 0.3 | 59.071 | 15.271 | 25.658 | 0.37:0.63 |
| 0.4 | 54.584 | 20.676 | 24.740 | 0.46:0.54 |
| 0.5 | 56.195 | 22.893 | 20.912 | 0.52:0.48 |
| 0.6 | 52.530 | 29.742 | 17.728 | 0.63:0.37 |

**Table S3.** XPS analysis of Ru 3d region of YMRO and YRO.

| **Species** | **YMRO** | | **YRO** | |
| --- | --- | --- | --- | --- |
|  | **Peak position (eV)** | **FWHM**  **(eV)** | **Peak position (eV)** | **FWHM**  **(eV)** |
| Ru 3d_3/2_ primary | 286.36 | 1.4 | 286.31 | 1.4 |
| Ru 3d_3/2_ satellite | 288.26 | 2.7 | 288.21 | 2.7 |
| Ru 3d_5/2_ primary | 282.08 | 1.5 | 282.03 | 1.7 |
| Ru 3d_5/2_ satellite | 283.98 | 2.8 | 283.93 | 2.7 |
| C 1s | 285.00 | 1.4 | 285.00 | 1.4 |

FWHM: Full width at half maximum.

**Table S4.** XPS analysis of Y 3d region of YMRO and YRO.

| **Species** | **YMRO** | | **YRO** | |
| --- | --- | --- | --- | --- |
|  | **Peak position (eV)** | **FWHM (eV)** | **Peak position (eV)** | **FWHM (eV)** |
| Y 3d_3/2_ primary | 158.49 | 1.2 | 158.54 | 1.2 |
| Y 3d_3/2_ secondary | 159.50 | 1.5 | 159.63 | 1.7 |
| Y 3d_5/2_ primary | 156.49 | 1.1 | 156.48 | 1.0 |
| Y 3d_5/2_ secondary | 157.38 | 0.9 | 157.15 | 1.4 |

**Figure S5**. XPS analysis of O 1s region of YMRO and YRO.

| **Species** | **YMRO** | | | **YRO** | | |
| --- | --- | --- | --- | --- | --- | --- |
|  | **Peak position (eV)** | **FWHM (eV)** | **Area (%)** | **Peak position (eV)** | **FWHM (eV)** | **Area (%)** |
| O 1s lattice oxygen | 528.96 | 1.2 | 46.9 | 529.04 | 1.2 | 38.1 |
| O 1s hydroxyl group | 531.10 | 2.6 | 44.4 | 531.12 | 2.8 | 56.1 |
| O 1s adsorbed water | 533.38 | 1.8 | 8.6 | 533.31 | 1.4 | 5.8 |
